# Acute Appendicitis Complicated by Superior Mesenteric Vein Thrombosis: A Rare Case of Pylephlebitis in a Healthy Young Male

**DOI:** 10.7759/cureus.88883

**Published:** 2025-07-28

**Authors:** Usamah Al-Anbagi, Hatem Abdulmajeed, Abdulqadir J Nashwan

**Affiliations:** 1 Internal Medicine, Hamad Medical Corporation, Doha, QAT; 2 Radiology, Hamad Medical Corporation, Doha, QAT; 3 Nursing and Midwifery Research, Hamad Medical Corporation, Doha, QAT

**Keywords:** acute appendicitis, antibiotics, anticoagulation therapy, computed tomography scan, mesenteric vein thrombosis, pylephlebitis

## Abstract

Pylephlebitis is a rare but potentially life-threatening complication of various intra-abdominal infections, characterized by septic thrombophlebitis of the portal venous system. It is most commonly associated with conditions such as appendicitis, diverticulitis, cholecystitis, and other sources of abdominal sepsis. When the thrombosis extends to the superior mesenteric vein, it can result in bowel ischemia, infarction, and even death if not promptly diagnosed and treated. We present the case of a 29-year-old man with no prior comorbidities who developed acute appendicitis complicated by thrombosis of the superior mesenteric and ileocolic veins. He underwent a successful laparoscopic appendectomy and was treated with anticoagulation therapy. The patient showed steady clinical improvement and was discharged on oral anticoagulation after a 12-day hospital stay, with complete recovery observed at follow-up visits conducted one week and two weeks after discharge. This case underscores the importance of early recognition of pylephlebitis in intra-abdominal infections and demonstrates that prompt surgical and anticoagulation management can lead to favorable outcomes.

## Introduction

Mesenteric vein thrombosis (MVT) is a rare but potentially life-threatening condition that can lead to bowel ischemia if not promptly diagnosed and treated. While MVT is typically associated with risk factors such as malignancy, intra-abdominal infections, recent surgery, and cirrhosis, its occurrence in the setting of acute or chronic appendicitis is exceptionally rare. Acute appendicitis remains one of the most common causes of abdominal pain requiring emergency surgery. In contrast, chronic or recurrent forms are less frequent, accounting for approximately 1% to 1.5% of cases, with some reports suggesting rates as high as 6.5% [1,2]. Although complications such as perforation and peritonitis are well recognized in acute appendicitis, superior mesenteric vein thrombosis (SMVT) is an uncommon and underreported complication. The nonspecific nature of SMVT symptoms poses a diagnostic challenge, potentially delaying treatment and increasing the risk of serious outcomes, including bowel infarction. However, advances in imaging, particularly contrast-enhanced CT scans, have significantly improved early detection and facilitated timely intervention. This case highlights a rare presentation of SMVT secondary to acute appendicitis in a young, previously healthy patient with no identifiable risk factors. The unusual involvement of both the superior mesenteric and the ileocolic veins emphasizes the importance of clinical vigilance to prevent life-threatening complications.

## Case presentation

A 29-year-old gentleman with no known past medical or surgical history presented to the emergency department with right lower quadrant (RLQ) abdominal pain that started three days before the presentation. The initial episode was severe and associated only with vomiting. The pain subsided over the next two days. However, he experienced a recurrence of severe RLQ pain on the day of presentation, which was not associated with vomiting. He denies any history of anorexia, constipation, diarrhea, or any other gastrointestinal symptoms. He also reports no urinary symptoms, though he noticed dark yellow urine. No other complaints were reported.

Upon examination, the patient was conscious and oriented, exhibiting mild distress, but was afebrile. Abdominal examination revealed a soft abdomen with tenderness in the RLQ, mild guarding, and positive rebound tenderness, without distension or rigidity; bowel sounds were present. The cardiovascular examination revealed normal heart sounds (S1, S2) with no murmurs. Neurologically, the patient had no deficits and no neck stiffness. The respiratory examination revealed equal bilateral air entry with no wheezing or crepitations.

The patient was examined in the emergency department and diagnosed with acute appendicitis. Initial laboratory investigations revealed elevated inflammatory markers along with mild liver function impairment (Table 1).

**Table 1:** Laboratory investigations ALP, alkaline phosphatase; ALT, alanine aminotransferase; APTT, activated partial thromboplastin time; AST, aspartate aminotransferase; CRP, C-reactive protein; Hb, hemoglobin; Hct, hematocrit; INR, international normalized ratio; PLT, platelet count; PT, prothrombin time; WBC, white blood cell

Parameter	On admission	Third day	On discharge	Reference values
Total leukocytes (×10³/µL)	10.3	13	10.5	4.0 × 10³/µL
Hct (%)	49.3	42	44	40-50
Hb (g/dL)	16.8	14.3	14.7	13-17
PLT (×10³/µL)	114	238	474	150-410
Serum urea (mmol/L)	11.1	3.8	-	2.5-7.8
Serum creatinine (µmol/L)	102	64	-	62-106
Serum potassium (mmol/L)	3.6	3.6	-	3.5-5.3
Serum sodium (mmol/L)	136	138	-	133-146
Serum total protein (g/L)	79	64	-	60-80
Serum albumin (g/L)	41	31	-	35-50
ALT (IU/L)	96	49	-	0-41
AST (IU/L)	81	30	-	0-41
ALP (U/L)	121	153	-	40-129
Serum total bilirubin (mg/dL)	51.9	14.4	-	0-21
CRP (mg/L)	448	255	-	0-5
PT (seconds)	13.5	25	23	9.4-12.5
INR	1.2	2.2	2	<1
APTT (seconds)	34.4	39.7	44.4	25.1-36.5

**Figure 1:**
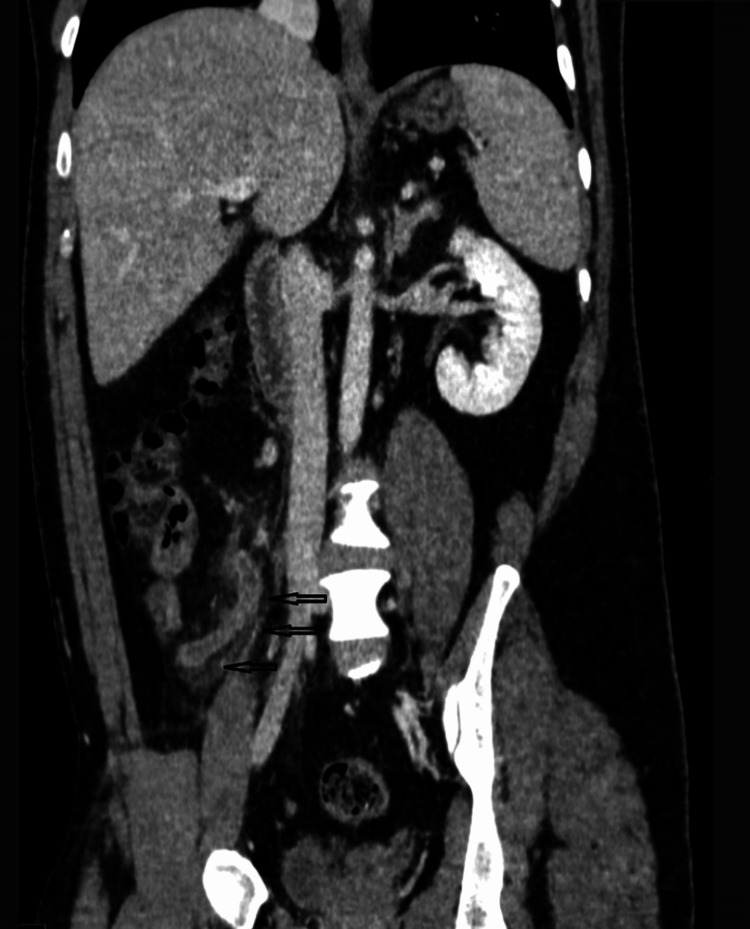
Coronal CT abdomen demonstrating features of acute appendicitis A CT scan of the abdomen in coronal view reveals inflammatory fat stranding surrounding the appendix. The black arrows indicate the appendix, with surrounding stranding consistent with acute appendicitis.

**Figure 2:**
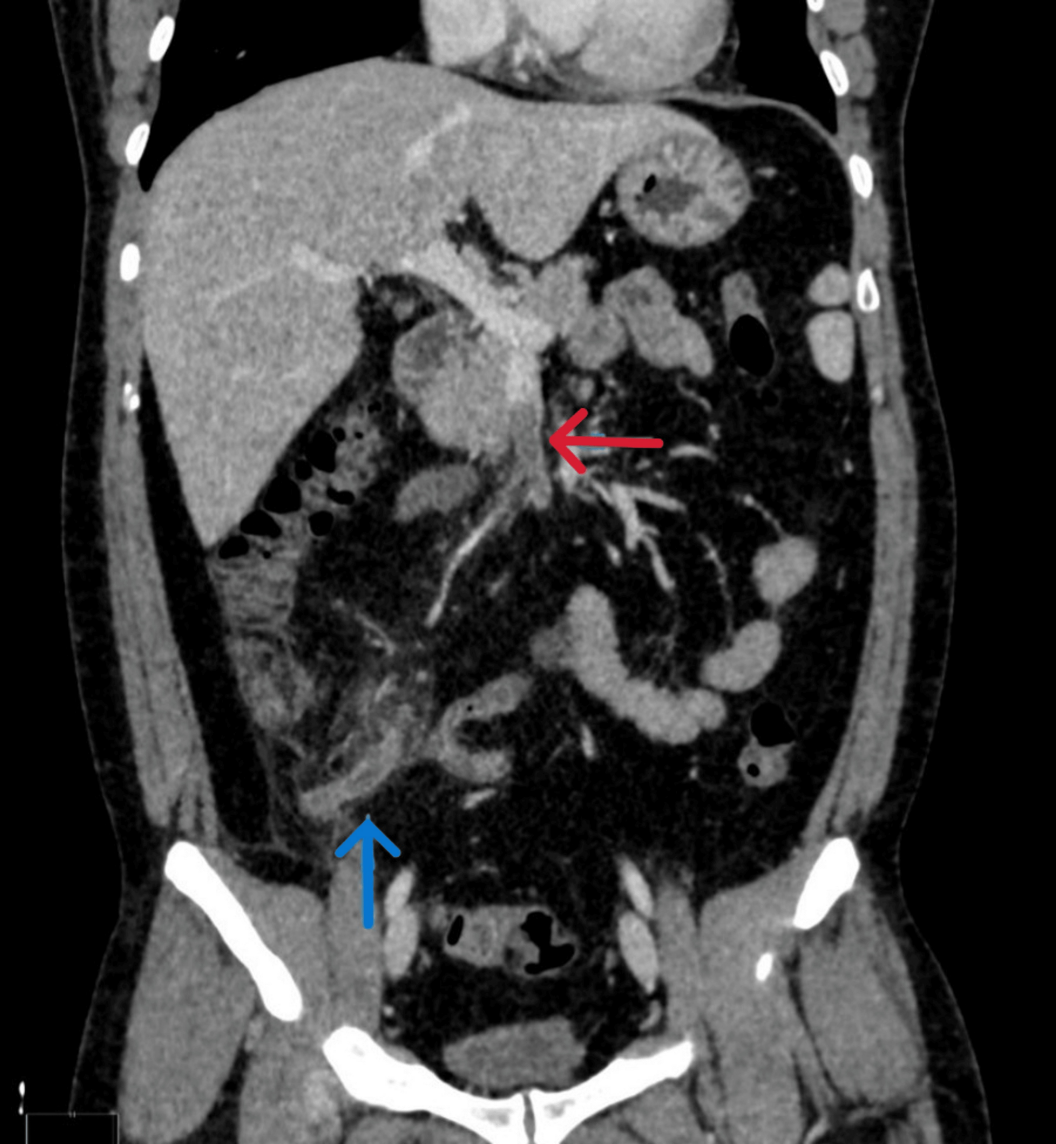
Coronal CT abdomen showing concurrent SMVT and acute appendicitis A CT scan of the abdomen in coronal view demonstrates a filling defect in the SMV, consistent with venous thrombosis (red arrow). The appendix appears inflamed with a thickened wall, suggestive of acute appendicitis (blue arrow). SMV, superior mesenteric vein; SMVT, superior mesenteric vein thrombosis

**Figure 3:**
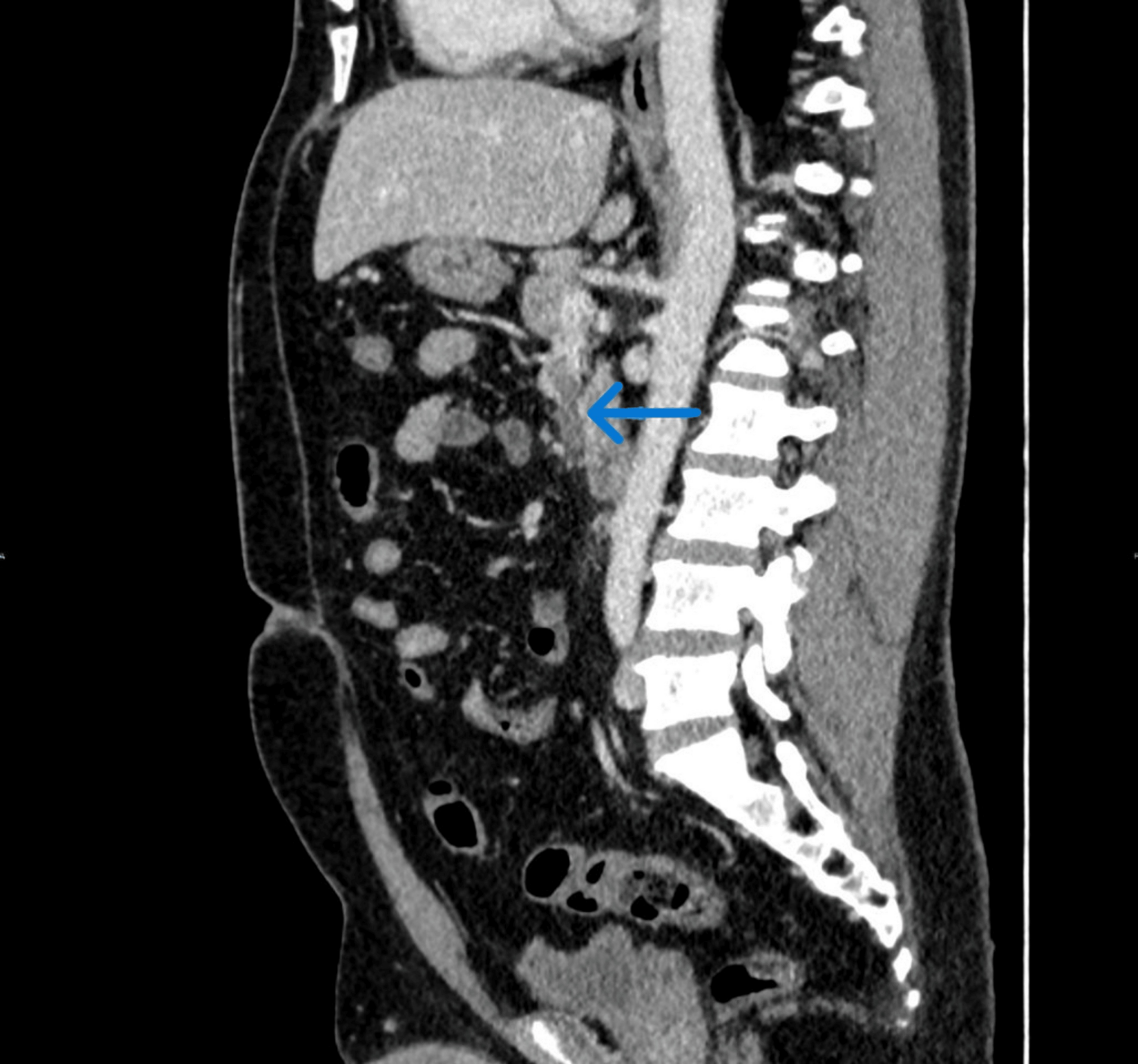
Sagittal CT abdomen showing SMVT A CT scan of the abdomen in sagittal view reveals a filling defect in the SMV, consistent with thrombosis. The blue arrow indicates the site of the thrombus. SMV, superior mesenteric vein; SMVT, superior mesenteric vein thrombosis

A CT scan of the abdomen was performed to confirm the diagnosis, which showed acute appendicitis (Figure 1, Figure 2), as well as an intraluminal thrombus within the lumen of the ileocolic vein and SMV, suggestive of venous thrombosis (Figure 2, Figure 3). He was admitted with a diagnosis of acute appendicitis complicated by venous thrombosis (pylephlebitis).

On the day following admission, the patient underwent a laparoscopic appendectomy. Hemostasis was secured. Postoperatively, he was started on a heparin infusion along with intravenous ampicillin-sulbactam 3 g every six hours. On day 3, the antibiotic was escalated to piperacillin-tazobactam 4.5 g every eight hours and continued for a total of seven days. The heparin infusion was transitioned to enoxaparin at a dose of 1 mg/kg/day.

The patient’s condition continued to show improvement, both clinically and in laboratory parameters, including a marked decline in C-reactive protein, liver enzymes (alanine aminotransferase and aspartate aminotransferase), and normalization of serum bilirubin level. He was discharged on warfarin 6 mg daily, which had been initiated three days prior, with enoxaparin used as bridging therapy. The plan was to continue anticoagulation for a total duration of six months, with a target international normalized ratio of 2-3 IU. He was followed up in the clinic after one week and again after two weeks. He remained clinically well, with complete resolution of his symptoms.

## Discussion

This case highlights a rare presentation of acute appendicitis complicated by SMVT and involvement of one of its tributaries, the ileocolic vein, in a previously healthy young male. The inflammatory response associated with appendicitis alone appeared sufficient to provoke thrombosis, underscoring the importance of considering vascular complications in patients presenting with atypical or severe abdominal pain, especially when imaging reveals venous involvement. Thrombosis in this case occurred in the setting of a clearly defined provoking factor, acute appendicitis. In alignment with current guidelines from the British Society for Haematology [3] and American Society of Hematology (ASH) [4], thrombophilia screening is not indicated in cases of provoked venous thromboembolism, as it does not influence management and may yield misleading results during acute illness. Early diagnosis, combined with the timely initiation of anticoagulation and surgical intervention, enabled successful treatment, resulting in complete clinical recovery and no recurrence on follow-up.

Appendicitis is a leading cause of acute abdominal pain and a common indication for emergency abdominal surgery worldwide. The anatomical position of the appendix can vary. Still, it is commonly retrocecal or pelvic and also located medially, laterally, anteriorly, or posteriorly to the cecum, leading to variations in clinical presentation [5]. Typically, patients experience periumbilical pain that migrates to the RLQ, accompanied by anorexia, fever, nausea, and vomiting. However, some may present with atypical or nonspecific symptoms such as bloating, indigestion, altered bowel habits, or generalized discomfort [6]. Diagnosis is often suspected based on RLQ tenderness and leukocytosis, but is confirmed by histopathological examination following surgical removal [7]. Complications of untreated or delayed appendicitis can be severe and include perforation, peri-appendiceal abscess, peritonitis, and sepsis. Perforation significantly increases the risk of widespread infection, while localized abscess formation may require drainage. Peritonitis and sepsis represent life-threatening consequences if the infection extends to the peritoneal cavity or bloodstream, underscoring the importance of early diagnosis and prompt surgical intervention.

Pylephlebitis is a rare but potentially life-threatening complication of intra-abdominal or pelvic infections, most commonly diverticulitis and appendicitis. It involves inflammation and septic thrombosis of the portal venous system [8]. Typically, it begins as thrombophlebitis of small veins at the site of infection, extending into larger vessels such as the portal and mesenteric veins. If not treated promptly, this progression can result in bowel ischemia, infarction, and death. Historically, the condition carried a near-universal fatality rate before the advent of antibiotics. A 2023 systematic review of cases from 1971 to 2022 identified diverticulitis (26.5%) and appendicitis (22%) as the leading causes [9]. Among affected vessels, the SMV was involved in up to 42% of cases [10], while the splenic vein and intrahepatic portal branches were less commonly affected.

SMVT remains an uncommon yet serious complication of various intra-abdominal inflammatory conditions, including but not limited to appendicitis and diverticulitis; other causes, such as pancreatitis, inflammatory bowel disease, and abdominal infections, should also be considered. It can lead to acute mesenteric ischemia, characterized by a sudden reduction in blood supply to the intestines resulting from obstructed venous outflow. MVT may present in acute, subacute, or chronic forms. It is classically associated with Virchow’s triad: blood stasis, endothelial injury, and a hypercoagulable state. The SMV, which drains the jejunum and ileum, is the most frequently involved site, whereas inferior mesenteric vein involvement is rare [11].

In acute venous occlusion, elevated venous pressure leads to bowel wall edema and hemorrhage, and if prolonged, ischemic infarction [12]. However, infarction is not universal, as some patients may develop collateral venous circulation, especially in chronic cases [13]. MVT is usually multifactorial, influenced by acquired risk factors such as pancreatitis, inflammatory bowel disease, intra-abdominal infections, and surgical trauma (e.g., splenectomy). Inherited or systemic hypercoagulable states, including myeloproliferative disorders, prothrombin gene mutations, nephrotic syndrome, and malignancy, are more likely to cause isolated mesenteric thrombosis [14,15]. The clinical presentation of MVT is often nonspecific and varies with disease acuity. In acute MVT, patients commonly experience colicky, periumbilical pain disproportionate to physical findings. In subacute cases, pain may be dull and persistent, lasting for days or weeks. Chronic MVT may be asymptomatic and detected incidentally or present with complications like portal hypertension, variceal bleeding, or postprandial pain in rare cases [13,16]. The drop in serum albumin from 35 to 31 g/L is consistent with an acute-phase reaction to inflammation. Hypoalbuminemia, even when mild, can promote vascular leakage and support a hypercoagulable state, which may have contributed to the development of SMVT in this case.

The cornerstone of treatment for acute and subacute MVT is conservative, with systemic anticoagulation to prevent thrombus extension and encourage recanalization [17]. Additional supportive measures include intravenous fluids, bowel rest, and close monitoring for signs of intestinal ischemia [18]. Although rare, endovascular or surgical interventions are reserved for cases that are refractory or complicated. Current guidelines recommend considering more aggressive approaches in the presence of hemodynamic instability, peritonitis, or radiologic evidence of bowel necrosis [19,20]. Anticoagulation should be continued for at least three to six months, with extended duration in those with ongoing risk factors [21]. Studies have demonstrated that anticoagulation significantly improves outcomes, reduces recurrence, and enhances vascular recanalization rates [16,21].

## Conclusions

This case exemplifies the critical importance of recognizing and managing rare complications, such as SMVT, in patients presenting with acute abdominal pain. Despite the absence of traditional risk factors, the inflammatory response from appendicitis alone can trigger vascular complications, necessitating a high degree of clinical suspicion. This case highlights the importance of vigilance in identifying vascular involvement in intra-abdominal infections, as early intervention can significantly improve patient outcomes and reduce morbidity and mortality. It also highlights the importance of increased clinical awareness and consideration of early imaging in appendicitis cases with atypical or severe features, which may guide timely diagnosis and appropriate management of such life-threatening complications.

## References

[1] Shah SS, Gaffney RR, Dykes TM, Goldstein JP (2013). Chronic appendicitis: an often forgotten cause of recurrent abdominal pain. Am J Med.

[2] Barber MD, McLaren J, Rainey JB (1997). Recurrent appendicitis. Br J Surg.

[3] Arachchillage DJ, Mackillop L, Chandratheva A, Motawani J, MacCallum P, Laffan M (2022). Thrombophilia testing: a British Society for Haematology guideline. Br J Haematol.

[4] Middeldorp S, Nieuwlaat R, Baumann Kreuziger L (2023). American Society of Hematology 2023 guidelines for management of venous thromboembolism: thrombophilia testing. Blood Adv.

[5] Williams GR (1983). Presidential address: a history of appendicitis. With anecdotes illustrating its importance. Ann Surg.

[6] Lee SL, Walsh AJ, Ho HS (2001). Computed tomography and ultrasonography do not improve and may delay the diagnosis and treatment of acute appendicitis. Arch Surg.

[7] Bessoff KE, Forrester JD (2020). Appendicitis in low-resource settings. Surg Infect (Larchmt).

[8] Kasper DL, Sahani D, Misdraji J (2005). Case 25-2005 — a 40-year-old man with prolonged fever and weight loss. N Engl J Med.

[9] Fusaro L, Di Bella S, Martingano P (2023). Pylephlebitis: a systematic review on etiology, diagnosis, and treatment of infective portal vein thrombosis. Diagnostics.

[10] Kanellopoulou T, Alexopoulou A, Theodossiades G (2010). Pylephlebitis: an overview of non-cirrhotic cases and factors related to outcome. Scand J Infect Dis.

[11] Abu-Daff S, Abu-Daff N, Al-Shahed M (2009). Mesenteric venous thrombosis and factors associated with mortality: a statistical analysis with five-year follow-up. J Gastrointest Surg.

[12] Johnson CC, Baggenstoss AH (1949). Mesenteric vascular occlusion; study of 99 cases of occlusion of veins. Proc Staff Meet Mayo Clin.

[13] Harnik IG, Brandt LJ (2010). Mesenteric venous thrombosis. Vasc Med.

[14] Salinas J, Barros D, Salgado N (2014). Portomesenteric vein thrombosis after laparoscopic sleeve gastrectomy. Surg Endosc.

[15] Villagrán R, Smith G, Rodriguez W (2016). Portomesenteric vein thrombosis after laparoscopic sleeve gastrectomy: incidence, analysis and follow-up in 1236 consecutive cases. Obes Surg.

[16] Amitrano L, Guardascione MA, Scaglione M (2007). Prognostic factors in noncirrhotic patients with splanchnic vein thromboses. Am J Gastroenterol.

[17] Brunaud L, Antunes L, Collinet-Adler S (2001). Acute mesenteric venous thrombosis: case for nonoperative management. J Vasc Surg.

[18] Singal AK, Kamath PS, Tefferi A (2013). Mesenteric venous thrombosis. Mayo Clin Proc.

[19] Stein M, Link DP (1999). Symptomatic spleno-mesenteric-portal venous thrombosis: recanalization and reconstruction with endovascular stents. J Vasc Interv Radiol.

[20] Patel IJ, Rahim S, Davidson JC (2019). Society of Interventional Radiology Consensus Guidelines for the periprocedural management of thrombotic and bleeding risk in patients undergoing percutaneous image-guided interventions—part II: recommendations
endorsed by the Canadian Association for Interventional Radiology and the Cardiovascular and Interventional Radiological Society of Europe. J Vasc Interv Radiol.

[21] Abdu RA, Zakhour BJ, Dallis DJ (1987). Mesenteric venous thrombosis--1911 to 1984. Surgery.

